# Changes in Antipsychotic Medication Adherence Among Medicaid Beneficiaries with Schizophrenia During COVID-19

**DOI:** 10.1007/s10488-024-01416-9

**Published:** 2024-10-11

**Authors:** Siyuan Shen, Catherine Yang, Molly Candon, Emily Lorenc, Min Jang, David Mandell

**Affiliations:** 1 Department of Psychiatry, Perelman School of Medicine, University of Pennsylvania, 3535 Market St, 3rd Floor, Philadelphia, PA 19104 USA; 2 Department of Health Care Management, Wharton School, University of Pennsylvania, Philadelphia, PA USA; 3 Leonard Davis Institute of Health Economics, University of Pennsylvania, Philadelphia, PA USA

**Keywords:** Telehealth, Schizophrenia, Medication adherence, COVID pandemic

## Abstract

To identify patterns of medication adherence during the pandemic and factors associated with these patterns among Medicaid-enrolled individuals with schizophrenia who had highly adherent medication use prior to the COVID-19 pandemic. We used Medicaid claims from Philadelphia to identify individuals with schizophrenia ≥ 18 years of age, their demographic characteristics, and health service use. We used group trajectory models to identify adherence trends, and ANOVA to examine associations between adherence groups and demographic characteristics and service use. The sample included 1,622 individuals. A 4-group trajectory model best fit our data. Seventy percent of individuals averaged about 92% adherence throughout the study period; 10% experienced a pronounced decline when the pandemic started (pandemic non-adherers); 11% experienced a sharp decline mid-pandemic (late non-adherers); and 9% experienced a sharp decline at the beginning of the pandemic and returned to higher adherence after a year (disrupted adherers). Adherers were least likely to be diagnosed with a substance use disorder, and had more telehealth visits, mental health outpatient visits, and fewer emergency department visits on average. Late non-adherers were more likely than adherers to have substance use disorders and physical health conditions. Pandemic non-adherers had more co-occurring psychiatric disorders than adherers and had the lowest use of case management. Three in ten previously adherent individuals with schizophrenia became less adherent to antipsychotic medications, either at the onset or later in the pandemic. Our findings point to telehealth and case management as critical strategies for treatment engagement, especially during public health crises, and well as the need to address co-occurring conditions.

## Introduction

Long-term use of antipsychotic medications in combination with psychosocial intervention is the established first line treatment for individuals with schizophrenia (Keepers et al., 2020). In addition to addressing symptoms, antipsychotic medications decreases risk of relapse, suicide attempts, rehospitalization, and mortality (Bodén et al., 2011; Keepers et al., 2020; Novick et al., 2010; Tiihonen et al., 2018).

Many care guidelines suggest that once individuals with schizophrenia are on a stable, effective dose of antipsychotic medication, they should meet with their prescriber at least monthly to assess symptoms and side effects (Exceptional Surveillance of Psychosis & Schizophrenia in Children, 2023; Keepers et al., 2020; Pringsheim et al., 2017). Unlike many other psychiatric conditions, medications to treat schizophrenia continue to be managed primarily by psychiatrists and psychiatric nurse practitioners in specialty mental health settings, rather than in primary care (Woodall et al., 2024).

The COVID-19 pandemic dramatically disrupted treatment for people with many chronic conditions, including schizophrenia (Muruganandam et al., 2020; Ruksakulpiwat et al., 2022). The social isolation accompanying the pandemic increased negative symptoms among people with schizophrenia, which may have reduced their motivation to participate in treatment (Strauss et al., 2022). Use of both inpatient and outpatient mental health services diminished dramatically during the pandemic because of concerns over the spread of COVID-19, which resulted in less contact with care teams (Busch et al., 2022; Zhu et al., 2022). The switch from in-person to telehealth in specialty mental health was more variable than, for example, in primary care (Cantor et al., 2021), and occurred less frequently for people with schizophrenia than for people with other psychiatric disorders (Bareis et al., 2023; Civan Kahve et al., 2021).

In light of these changes, it is not surprising that adherence to antipsychotics among people with schizophrenia also decreased. In their interview study of 800 patients in China, Yao and colleagues found that 41.5% reported that they were adhering to their medications during the pandemic (Yao et al., 2022). In a similar survey study, Wang and colleagues reported adherence rates of 51.5% (Wang et al., 2023). Both studies were cross-sectional, relied on self-reports, and neither had data on medication adherence prior to the pandemic. A third study using Medicare claims found a 20% reduction in medication fills for people with severe mental illness (Busch et al., 2022). This longitudinal study did not examine factors associated with changes in medication use, including whether particular groups were more vulnerable than others.

Understanding factors associated with non-adherence has important implications for identifying people who may now need additional supports, preparing for future disruptions to the mental health care system, and identifying protective factors that can be replicated. In the present study, we used group trajectory analysis (Alhazami et al., 2020; Franklin et al., 2013; Hickson et al., 2020; MacEwan et al., 2016; Nagin & Odgers, 2010) to identify patterns of antipsychotic use during the pandemic among Medicaid-enrolled individuals with schizophrenia who were highly adherent to their medication prior to the pandemic. We then examined factors associated with membership in the adherence groups.

## Methods

### Data Source

We used Medicaid enrollment files from the Philadelphia’s Department of Behavioral Health and Intellectual disAbility Services (DBH) to identify Medicaid enrollees who met eligibility requirements during the 2019–2021 study period. We used Medicaid behavioral health claims to identify people with a diagnosis of schizophrenia at any time during the 2019 calendar year. We used the Medicaid pharmacy claims to estimate antipsychotic medication adherence over time. We used enrollment files, behavioral health claims, and physical health claims to examine factors associated with each trajectory of adherence.

### Sample

The sample included Medicaid-enrolled individuals, ages 18 to 63 years in 2019, who were continuously enrolled for 3 years, who met two criteria: (1) they had at least one claim associated with a diagnosis of schizophrenia (ICD-10 code F20) (The ICD, 2023) in 2019; and (2) they had a prescription for antipsychotic medication in 2019, and had at least 80% proportion of days covered (PDC) during 2019. Members who were institutionalized for > 90 days in 2019 were excluded (Korhonen et al., 2016; Lo-Ciganic et al., 2016). Days spent in an institution were removed prior to PDC calculation (Valenstein et al., 2002).

### Variables of Interest

We defined medication adherence using proportion of days covered (PDC). We constructed a record for each study participant using PDC to represent daily medication coverage over a 3-year period. We estimated quarterly adherence as number of days with antipsychotic medication supply during each quarter.

Baseline demographic characteristics were extracted from the Medicaid enrollment files and included age as of December 31, 2019, race/ethnicity (non-Hispanic Asian, non-Hispanic Black, Hispanic, non-Hispanic White, and other), and sex (male, female). Clinical characteristics included both behavioral health and physical health conditions during the 6-month pre-period, which was the 3rd and 4th quarter of 2018. We used clinical classifications software (CCS) (CCS, 2023) from the Agency for Healthcare Research and Quality to identify behavioral health conditions and Elixhauser comorbidity indices refined for ICD-10-CM (Elixhauser, 2023) to identify the most common physical health conditions. Whether the participant received Social Security income (SSI) was identified through the Medicaid enrollment file, again during the 6-month pre-period. Service use history, including emergency department (ED) admissions, medication management, case management, and inpatient psychiatric hospitalizations were identified through the place of service variable from physical health claims and level of care variable from behavioral health claims during the pre-period.

We examined whether medication adherence was associated with mental health outpatient visits, telehealth visits, or ED visits between 2019 and 2021. We created these variables using the place of service variable from both physical health and behavioral health claims and measured quarterly. We also categorized antipsychotics by whether they were long-acting injectables (LAI), which we identified using the drug dosage ‘HH’, ‘HQ’, ‘VR’ in the pharmacy claims.

### Statistical Analysis

To estimate patterns of adherence, we used a group-based trajectory model, using ‘Proc Traj’ (A SAS, 2001), an add-on module to SAS for identifying longitudinal trajectories (Twisk & Hoekstra, 2012). We tested models with 2, 3, 4, 5 and 6 groups. Based on these models, we estimated the probability of membership in each group and assigned patients to the trajectory group with the highest membership probability (A SAS, 2001; Franklin et al., 2013) We used the Bayesian Information Criterion (BIC) statistic and clinical judgement to select the model with the optimal number of trajectory groups to describe the data (Nagin & Odgers, 2010).

We used ANOVA tests to estimate the associations between each demographic characteristic, behavioral and physical health condition, treatment history, and 3-year PDC among the four groups. To examine associations between adherence group and service use, we used a mixed-model repeated-measures analysis of covariance. We used a split-plot design to track the effect of time, group, and the interaction between group and time.

## Results

A total of 1622 individuals met selection criteria. They were mostly male (64.9%) with an average age of 45.6 years; 69% were Black, 9% Hispanic, 13% White, and 6% Asian. Most (87%) were Medicaid eligible through the disability category.

**Fig. 1:**
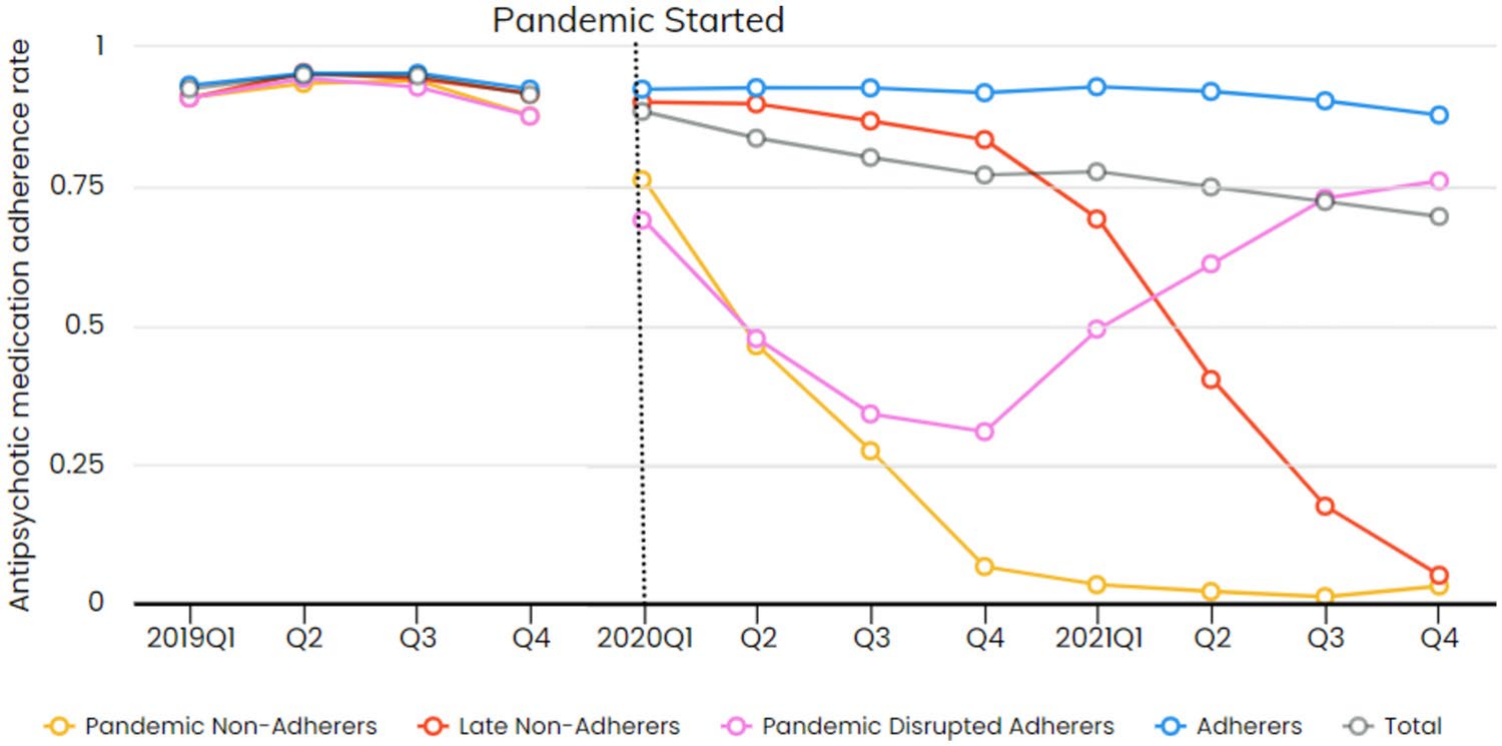
Subgroup trends in antipsychotic medication adherence during COVID-19, 2019–2021

**Fig. 2:**
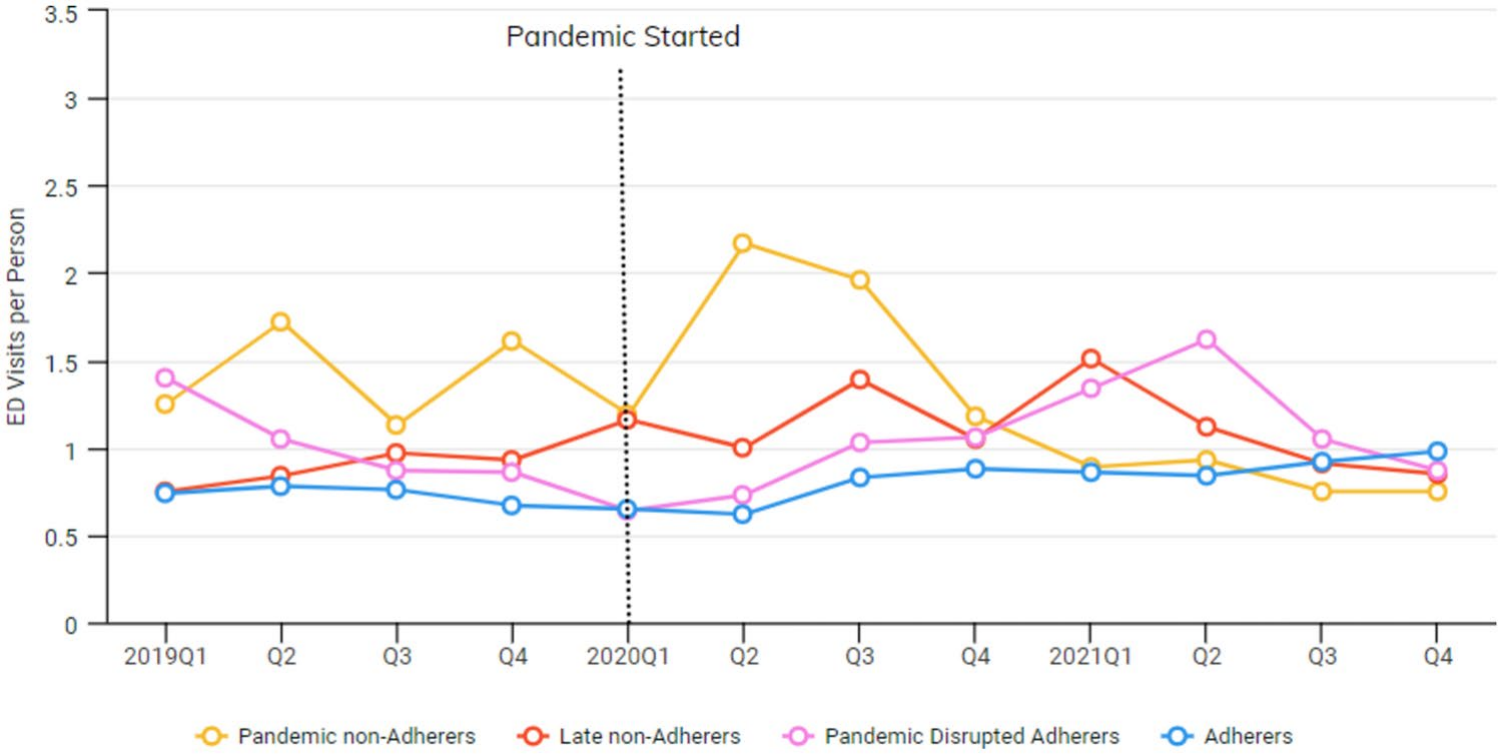
Avg. number of ED visits per person by adherence groups

**Fig. 3:**
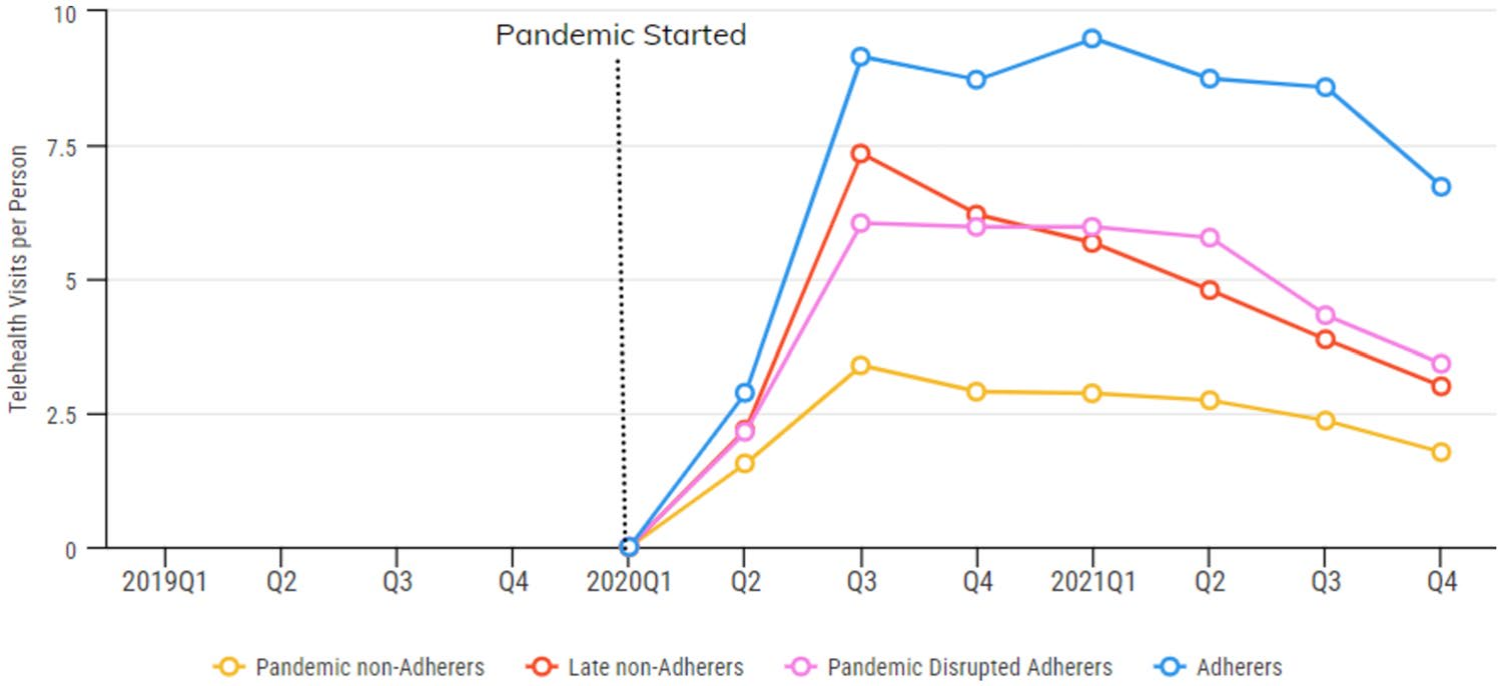
Avg. number of telehealth visits per person by adherence groups

**Fig. 4:**
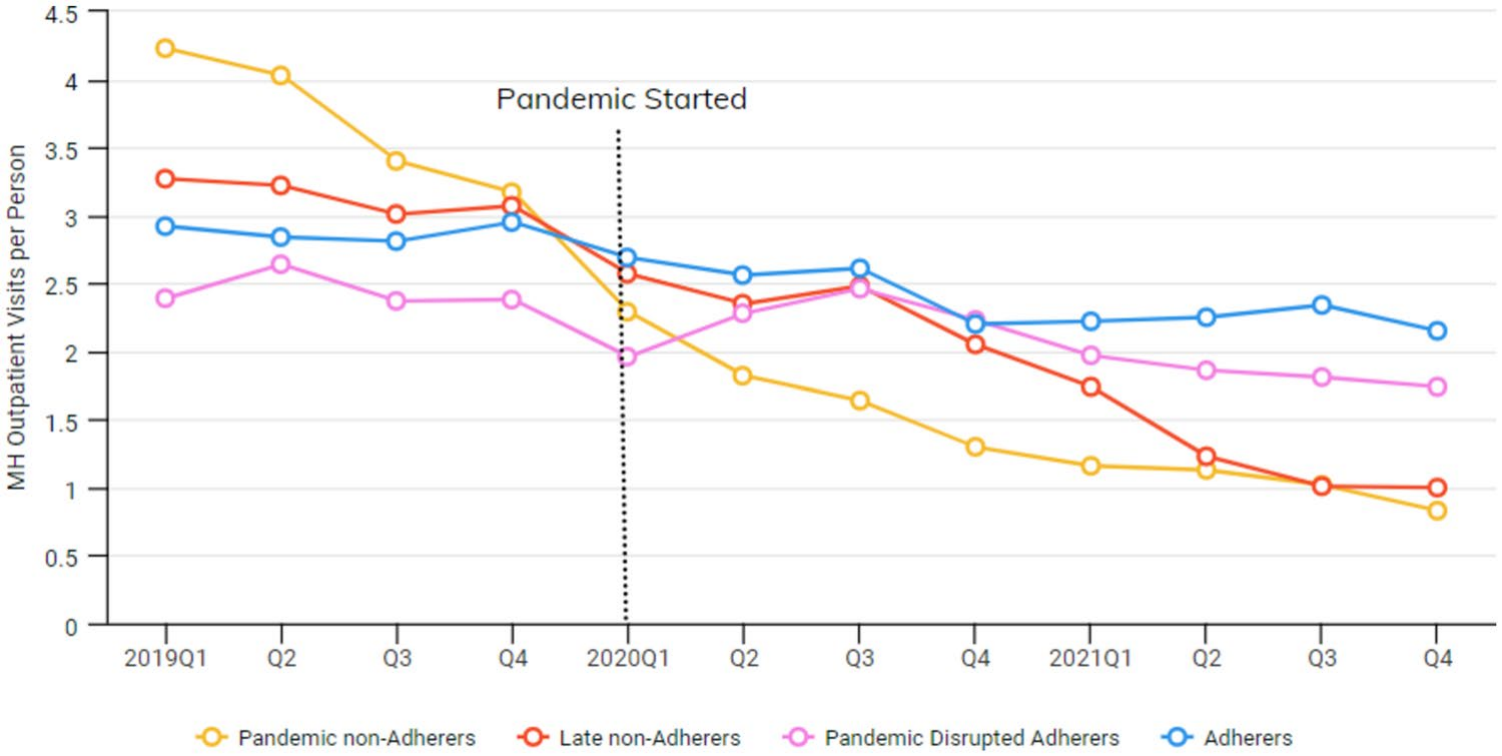
Avg. number of MH Outpatient visits per person by adherence groups

A 4-group trajectory model best fit our data (Fig. 1). Ten percent of the sample experienced a pronounced decline in adherence when the pandemic started and remained non-adherent for the rest of the study period (pandemic non-adherers). Eleven percent adhered until 2021 and then experienced a steep decline (late non-adherers). Nine percent experienced a decline when the pandemic started and then increased adherence in 2021 (pandemic disrupted adherers). Most participants (70%) averaged about 92% adherence throughout the study period (adherers) (Figs. 2, 3 and 4).

Demographic characteristics and co-occurring conditions for each group are shown in Table 1. Adherers were least likely to be diagnosed with a substance use disorder and had fewer ED visits on average than other groups during the pre-period. Late non-adherers were similar to adherers, except they were more likely to have co-occurring substance use disorders and physical health conditions. Pandemic non-adherers were more likely to have co-occurring substance use disorders than adherers, while disrupted pandemic adherers had higher rates of psychiatric conditions, including depression.

**Table 1:** Characteristics of sample by medication adherence group (*n* = 1622)

	Pandemic non-Adherers	Late non-Adherers	Pandemic Disrupted Adherers	Adherers	*p* value
Group membership likelihood	97.7%	96.4%	94.1%	99.1%	
*N*	166	171	146	1,139	
%	10.2%	10.5%	9.0%	70.2%	
Demographic characteristics					
Black	78.9%	70.2%	77.4%	66.8%**	< 0.01
White	10.8%	12.9%	10.3%	14.1%	0.433
Hispanic	8.4%	9.0%	7.5%	9.9%	0.773
Asian	0.6%	4.1%*	2.7%	6.9%**	< 0.01
Male	63.3%	69.6%	62.3%	64.8%	0.517
Avg. age in 2019 [std]	46.4 [11.7]	46.1 [12.7]	44.7 [12.0]	45.5 [12.2]	0.623
SSI	88.6%	82.5%	84.9%	88.1%	0.161
Other psychiatric diagnoses					
Depressive disorders	15.7%	12.9%	15.8%	10.5%	0.089
Bipolar disorders	11.5%	5.9%	12.3%	8.4%	0.124
Opioid related disorders	4.8%	2.9%	2.1%	2.8%	0.474
Alcohol related disorders	9.0%	5.3%	8.2%	2.8%***	< 0.001
Other substance use disorders	21.7%	15.8%	25.3%	12.8%**	< 0.001
Physical comorbidities					
Hypertension	23.5%	28.7%	24.7%	26.1%	0.727
Diabetes	15.7%	19.3%	16.4%	16.7%	0.816
Chronic pulmonary disease	16.9%	17.0%	7.5%*	11.1%*	< 0.01
Obesity	23.5%	21.6%	22.6%	21.0%	0.879
Peripheral vascular disease	2.4%	2.3%	2.7%	2.4%	0.994
Prior service use					
ED admission	34.9%	25.2%*	37.0%	27.3%*	0.017
Medication management	48.8%	42.1%	44.5%	43.2%	0.556
Case management	21.1%	28.1%	26.0%	25.3%	0.513
Inpatient psychiatric admission	18.1%	12.3%	21.2%	12.0%*	< 0.01
Service use over the study period					
Avg. quarterly ED admission	1.3 [4.3]	1.0 [3.3]	1.0 [1.6]	0.8 [2.7]	0.154
Avg. quarterly MH outpatient visits	2.2 [3.4]	2.3 [3.6]	2.2 [3.0]	2.5 [4.5]	0.499
Avg. quarterly telehealth visits	2.2 [5.6]	4.1 [8.5]*	4.2 [6.3]**	6.8 [10.9]***	< 0.001
Medication form of delivery					
Long-acting Injectable	12.7%	29.8%***	35.6%***	21.0%*	< 0.001
Avg. proportion of Long-acting Injectable	6.3%	15.1%***	18.0%***	9.2%	< 0.001
PDC over the study period					
Mean PDC	45.0%	71.6%***	68.0%***	92.5%***	< 0.001
20–40%	27.1%	0.0%***	0.7%***	0.0%***	< 0.001
40–60%	71.1%	8.8%***	21.9%***	0.0%***	
60–80%	1.8%	76.0%***	71.9%***	3.3%	
> = 80%	0.0%	15.2%***	5.5%**	96.8%***	

Group Membership Likelihood measures the proportion of a population that follows a particular trajectory group

Physical Health data and Elixhauser Index were used to identify the top conditions for physical comorbidities

Pair-wise *t* test or Chi-square result (vs. Pandemic Non-Adherers: *p < 0.05; **p < 0.01; ***p < 0.001

Mixed-model analyses revealed significant variation among the four groups in the types of services they were likely to use. The adherers group had more mental health outpatient visits and telehealth visits than the other groups during the pandemic. Adherers were less likely to have an ED visit or hospitalization and were more likely to have case management visits. Pandemic non-adherers had the lowest rates of case management use and had more ED visits on average. Late non-adherers and pandemic disrupted adherers had the highest rate of LAI use.

## Discussion

We estimated changes in antipsychotic medication adherence following the start of the COVID-19 pandemic among persons with schizophrenia who were seen in Philadelphia's public mental health system and had high medication adherence to antipsychotics prior to the pandemic. While most of these individuals continued to have high antipsychotic adherence, 30% experienced a disruption in their receipt of antipsychotics following the start of the pandemic. Individuals whose medication adherence decreased were less likely to transition from in-person care to telehealth and less likely to receive case management services and were more likely to visit the ED and to be hospitalized.

This finding stands in contrast to a prior study, which found that reduced outpatient and inpatient visits were not associated with reduced adherence (Wilcock et al., 2023), although the sample for that study included only Medicare beneficiaries. The fact that 70% remained adherent to their medication regimen highlights both their personal strength and the adaptability of the Philadelphia publicly funded treatment system, which implemented creative and flexible approaches to care, including a capitated payment that maintained the service system and offered agencies flexibility in providing care.

Another 11% of our sample initially showed high adherence to medications, but steeply reduced their antipsychotic use about 12 months into the pandemic. It is worth reiterating that this group was highly adherent prior to the pandemic. The greater prevalence of LAI antipsychotics in the late non-adherers group might be one reason why their adherence did not decrease immediately following the start of the pandemic. Late non-adherers also showed declining use of telehealth over time, which suggests that telehealth may not have been an altogether acceptable substitute for in-person visits in this group.

Notably, ED visits, hospitalizations, and substance use disorders were common in the entire sample, highlighting the urgent need to provide a holistic set of supports to individuals with schizophrenia. Telehealth emerged as a critical tool in promoting medication adherence, particularly during times of crisis, and perhaps mitigated the types of events that result in hospitalization and ED use.

Those who stopped or reduced antipsychotic use during the pandemic were more likely to have a co-occurring psychiatric diagnosis than those in the adherer group, particularly depression and substance use disorders. Studies have shown that substance use increased during the pandemic, particularly among minoritized communities (Chacon et al., 2021) and among those with anxiety and depression (Capasso et al., 2021; Grossman et al., 2020; White et al., 2020). People with schizophrenia who experience these additional challenges may have been particularly vulnerable to non-adherence.

This study had several limitations, including our focus only on one city, the lack of clinical data on participants, and our correlational study design. Despite these limitations, there are important implications related to these findings. The pandemic may have resulted in as many as 3 in 10 individuals with schizophrenia becoming less adherent to their medications, either at the onset or later in the pandemic. While our results are correlational, they point to telehealth as one strategy to continue treatment engagement, similar to what prior studies have found (Cantor et al., 2021; Creedon et al., 2020). Telehealth could pair nicely with other technological innovations to increase adherence and engagement, including monitoring and text messaging, both of which have evidence to support their use (Byerly et al., 2007; Schulze et al., 2019; Simon et al., 2022).

While any discussion of the effects of telehealth during the pandemic based on this study are speculative, it is clear that, in the event of a similar disruption of care, we will need rapid and innovative strategies to ensure that individuals with schizophrenia receive the care they need.
